# Ba_1−x_Sr_x_Zn_2_Si_2_O_7_ - A new family of materials with negative and very high thermal expansion

**DOI:** 10.1038/srep18040

**Published:** 2015-12-15

**Authors:** Christian Thieme, Helmar Görls, Christian Rüssel

**Affiliations:** 1 Chair of Glass Chemistry I, Jena University, Fraunhoferstr. 6, 07743 Jena, Germany; 2 Institute of Inorganic and Analytical Chemistry, Jena University, Lessingstr. 8, 07743 Jena, Germany

## Abstract

The compound BaZn_2_Si_2_O_7_ shows a high coefficient of thermal expansion up to a temperature of 280 °C, then a transition to a high temperature phase is observed. This high temperature phase exhibits negative thermal expansion. If Ba^2+^ is successively replaced by Sr^2+^, a new phase with a structure, similar to that of the high temperature phase of BaZn_2_Si_2_O_7_, forms. At the composition Ba_0.8_Sr_0.2_Zn_2_Si_2_O_7_, this new phase is completely stabilized. The crystal structure was determined with single crystal X-ray diffraction using the composition Ba_0.6_Sr_0.4_Zn_2_Si_2_O_7_, which crystallizes in the orthorhombic space group Cmcm. The negative thermal expansion is a result of motions and distortions inside the crystal lattice, especially inside the chains of ZnO_4_ tetrahedra. Dilatometry and high temperature X-ray powder diffraction were used to verify the negative thermal expansion. Coefficients of thermal expansion partially smaller than −10·10^−6^ K^−1^ were measured.

Most of all materials expand during heating^1^,^2^,^3^. There are just a few silicates, exhibiting negative thermal expansion, such as beta-quartz and beta-eucryptite^4^,^5^,^6^. Besides, ZrW_2_O_8_ is one of the most commonly known negative thermal expansion (NTE) materials exhibiting a coefficient of thermal expansion of −8.7·10^−6^ K^−1^ 7,8. By contrast, barium silicates, normally exhibit extremely high coefficients of thermal expansion^9^. The compound BaZn_2_Si_2_O_7_ shows a high coefficient of thermal expansion up to a temperature of 280 °C, then a transition to a high temperature phase is observed, running parallel to a steep increase in volume of around 2%^10^. Below the phase transition, where the phase has a monoclinic structure with the space group C2/c, the mean value of the coefficient of thermal expansion is around 17.6·10^−6^ K^−1^ 11,12. Above this phase transition, the phase has the orthorhombic space group Ccm2_1_ and very low and partially negative thermal expansion is observed^11^,^12^. The stabilization of this high temperature phase down to room temperature was not possible until now. We found a new family of materials with giant negative coefficients of thermal expansion, partially smaller than −10·10^−6^ K^−1^, in solid solutions of the system Ba_1−x_Sr_x_Zn_2_Si_2_O_7_. If Ba^2+^ is successively replaced by Sr^2+^, a new phase with a structure, similar to that of the high temperature phase of BaZn_2_Si_2_O_7_, forms. At the composition Ba_0.8_Sr_0.2_Zn_2_Si_2_O_7_, this new phase is completely stabilized and can easily be crystallized from silicate glasses which is of great importance because all commercial available zero expansion materials are glass-ceramics. To the best of our knowledge, it is the first new silicate phase with negative thermal expansion reported in the past 50 years. In comparison to alumosilicates, the manufacturing process should be much easier.

Ceramics and glass-ceramics containing BaZn_2_Si_2_O_7_ and other isostructural compounds are well known as high thermal expansion materials^10^,^11^,^12^,^13^. The substitution of Zn^2+^ by other ions with similar ionic radii and the same valence state, such as Co^2+^, Mg^2+^ or Ni^2+^, leads to a shift of the phase transition towards higher temperatures. Klasens *et al.* reported already in 1957^15^ that the substitution of BaO by SrO leads to the formation of a new phase with unknown crystal structure. They also reported that a complete replacement of BaO by SrO is not possible. Up to now, this phase was not further studied, especially it was not discovered that this phase has a negative thermal expansion.

In Fig. 1, the crystal structure of the Ba_0.6_Sr_0.4_Zn_2_Si_2_O_7_ phase, determined using single crystal X-ray diffraction is shown in the left part of the image in four different views. The samples were prepared from a stoichiometric melt with a BaO:SrO-ratio of 1:1, but the refinement of the crystal structure showed that the composition of the investigated single crystal is Ba_0.6_Sr_0.4_Zn_2_Si_2_O_7_. This composition was also confirmed via energy-dispersive X-ray spectroscopy (EDX). The reason for this difference should be a not completely homogenized melt from which these single crystals were prepared. A preferred incorporation of Ba^2+^ into the structure is not assumed because of the much higher ionic radius in comparison to Sr^2+^.

The crystal structure information as well as the refinement parameters are summarized in Table 1. Silicon as well as zinc are fourfold coordinated. Two SiO_4_ tetrahedra are bridged by an oxygen. In analogy, also the ZnO_4_ tetrahedra are bridged by oxygen. In these tetrahedra, two of four oxygens act as bridges between two ZnO_4_ tetrahedra and one SiO_4_ tetrahedron. The other two oxygens bridge only two silicon atoms. The Ba- and Sr- atoms are surrounded by 5 oxygens, forming a pyramid with a quadratic basal plane. The right part of Fig. 1 shows a comparison of the b-c-plane of Ba_0.6_Sr_0.4_Zn_2_Si_2_O_7_ and the a-c-plane of HT-BaZn_2_Si_2_O_7_. The image of the latter was calculated from the crystal structure information of HT-BaZn_2_Si_2_O_7_ measured at 350 °C in ref. 14. The different definitions of the unit cells and lattice parameters of both phases make it necessary to compare different planes and directions as it is done in Fig. 1 for the b-c- and the a-c-planes. It can be seen that there are similar atomic arrangements in both phases (lower part on the right side of Fig. 1). The differences in the crystal structures of both phases are only marginal so that both phases should exhibit similar thermal expansion properties.

In Fig. 2, X-ray powder diffraction patterns in the solid solution series Ba_1-x_Sr_x_Zn_2_Si_2_O_7_ with different values of x, measured at room temperature, are shown. It can be seen that the crystal structure changes from x = 0 to x = 0.1. The diffractogram with x = 0 is that of pure LT- BaZn_2_Si_2_O_7_. In the diffraction pattern with x = 0.1, it can be seen that the new crystal phase has formed. Minor concentrations of crystals with the structure of LT-BaZn_2_Si_2_O_7_ were also found. At higher x-values, i.e. higher Sr-concentrations, the new structure is completely stabilized and hence, the only occurring. The peak positions for 0.1 ≤ × ≤ 0.9 are approximately in agreement with those from the single crystal measurement and those from ref.^15^ (see the lower two patterns on the left side of Fig. 2). The peak positions of the respective graphs are somewhat shifted; the introduction of more strontium into the lattice results in somewhat larger 2θ values, i.e. in smaller lattice constants, which can be clearly seen by comparing the samples with x = 0.1 and 0.9. This can easily be explained by the smaller cation radius of Sr^2+^. A complete substitution of BaO by SrO does not result in single phase materials but in a mixture of crystals with the structure of HT-BaZn_2_Si_2_O_7_, together with Sr_2_ZnSi_2_O_7_ and Zn_2_SiO_4_. However, in the range of 0.1 ≤ × ≤ 0.9 single phase materials with the crystal structure similar to HT-BaZn_2_Si_2_O_7_ were obtained.

The change in the crystal structure runs parallel to an exceptional change in the thermal expansion behavior. This can be seen in the right part of Fig. 2. The sample with x = 0 exhibits extremely high thermal expansion behavior (the data were taken from ref. 10). At around 280 °C, a phase transition leads to the formation of the HT-modification, which shows partly negative thermal expansion. The samples with x = 0.1 and 0.8, where a crystal structure similar to HT-BaZn_2_Si_2_O_7_ is stable, show extremely low thermal expansion behavior, which is comparable to that of the sample with x = 0 above around 300 °C. It should be mentioned that in the case of NTE materials, the thermal expansion behavior measured by dilatometry, strongly depends on the sample preparation and microstructure 16,17. The sintered ceramic bodies showed strong hysteresis behavior, which might be affected by micro cracking. However, all samples with the structure of HT-BaZn_2_Si_2_O_7_ exhibit negative thermal expansion. A correlation of the dilatometric thermal expansion and the SrO concentration was not found.

A contraction during heating can also be seen in Fig. 3, which pictorially presents the changes in the crystal structure with temperature. The upper part of the picture (A) shows a rotational movement of ZnO_4_ tetrahedra inside a ZnO_4_ chain. This rotation in general leads to a strong increase in length in the direction of the chain (see the length, denoted as “x” in Fig. 3A) as well as a small increase perpendicular to this direction (y). However, as it is illustrated by the dashed lines, the rotation of the tetrahedra also leads to a strong shortening of the distance z. In the direction perpendicular to the chain (b-axis), the ZnO_4_ tetrahedra are connected with rigid SiO_4_ tetrahedra (Fig. 3B). A rotation inside the chain now leads to the effect that some of the SiO_4_ tetrahedra are pulled near the chain and other are slightly pushed away. As it is illustrated in Fig. 3A, the effect of pulling, i. e. shortening in the direction of the b-axis, is much more pronounced than the contrary effect. This leads to strong distortions of the tetrahedra, which are much more pronounced for the ZnO_4_ tetrahedra, which will be strongly compressed, perpendicular to the chain and can be seen in Fig. 3C in the a-b-plane of the crystal. These results are based on single crystal X-ray diffraction measurements. On the left side, the crystal structure is displayed. On the right side, the distortions of the SiO_4_ and the ZnO_4_ tetrahedra are shown for different temperature ranges. It can be clearly seen that the distortion of the ZnO_4_ tetrahedra is much stronger than that of the SiO_4_ tetrahedra. This distortion, together with small tilting motions of the tetrahedra lead to an obvious decrease in the O1–O1 distance of 0.652% between −55 and 80 °C. This is the most pronounced contraction found in this crystal structure. Furthermore, the rotation of ZnO_4_ units also leads to a change in bond angles away from that of an ideal tetrahedron. As it is demonstrated in Fig. 3B, the SiO_4_ tetrahedra will be stretched in the direction of the chain, which can be seen by the increase of the respective O-Si-O angle, which increases from 115.778 at −55 °C to 115.921 at 80 °C.

Furthermore, the rotational movement of ZnO_4_ tetrahedra will also explain the highly anisotropic thermal expansion behavior with coefficients of thermal expansion of 9.5∙10^−6^ K^−1^ (a-axis), −32.1∙10^−6^ K^−1^ (b-axis), and 23.1∙10^−6^ K^−1^ (c-axis) (measured between 30 and 600 °C). The highest thermal expansion can be found in the direction of the ZnO_4_ chains and negative thermal expansion perpendicular to the chain in the direction of the b-axis. The thermal expansion in the direction of the a-axis does not seem to be affected by the motions of the chains. This reason can be found in the left part of Fig. 1, where the a-c-plane is shown. There it can be seen that the ZnO_4_ chains are highly oriented in this plane so that a motion of the chains in these directions should be energetically not favored.

Figure 4 shows the thermal expansion behavior of Ba_0.6_Sr_0.4_Zn_2_Si_2_O_7_. Powdered specimens of Ba_0.6_Sr_0.4_Zn_2_Si_2_O_7_ were studied using high temperature powder X-ray diffraction. For a better illustration, Fig. 4 shows the powder XRD-patterns for a narrow 2θ-range from 18 to 42° (left part of the picture). To enable a more accurate determination of the 2θ-values, the powder was mixed with Al_2_O_3_ as internal standard. Furthermore, the peak positions of the Ba_0.6_Sr_0.4_Zn_2_Si_2_O_7_ phase and Al_2_O_3_, taken from the single crystal measurement and Ref. 18are shown. It is seen that the peak at around 27° is shifted to larger 2θ values with increasing temperature, which shows that the corresponding lattice spacings decrease with increasing temperature. This peak can be attributed to the (220) lattice plane. In the upper right corner of Fig. 4, the cell parameters, calculated from these XRD measurements, are shown as a function of temperature. A strong contraction of the lattice parameter of the b-axes with increasing temperature is obvious. This effect leads to the contraction of the complete unit cell.

However, as mentioned above, the coefficient of thermal expansion of the pure crystalline ceramics strongly depends on the sample preparation. The strong anisotropic thermal expansion behavior may lead to the formation of cracks, additionally lowering the coefficient of thermal expansion. Hence, the thermal expansion behavior determined via HT-XRD measurements, is the more reliable and might differ strongly from dilatometric results. An illustration of the NTE behavior of samples with the composition Ba_0.5_Sr_0.5_Zn_2_Si_2_O_7_ prepared at different temperatures and sintering conditions is shown in the lower right corner of Fig. 4, where the coefficient of thermal expansion in a temperature range between 100 and 800 °C varies between −4.3 and −14.8∙10^−6^ K^−1^. The strong variation of the thermal expansion behavior of bulk ceramic samples in dependence of the preparation method is commonly known and the fabrication of dense sintered and crack-free NTE materials is highly challenging^19^,^20^. This problem is avoided by crystallization of such phases from glasses.

Crystalline phases with negative thermal expansion coefficients are not only suitable for the production of materials with a coefficient of thermal expansion, which is negative or close to zero, but can also be used for a large variety of other purposes. Among these are:

- Materials composed by a bulk material with positive thermal expansion and a surface layer of a material with negative thermal expansion. During cooling of such a material, compressive stresses at the surface are formed which might result in high strength materials.
- Lamellar structures composed of alternating layers of materials with positive and negative thermal expansion. Also here, stresses are formed during cooling which should especially result in high fracture toughness.
- Joining of materials with low thermal expansion, such as vitreous silica or Ceran^©^ Glass Ceramics. The seal has an adapted thermal expansion coefficient and can be sealed at temperatures notably below the softening point of e.g. Ceran^©^ or vitreous silica. Such seals up to now are not available.
- Sealing Materials with adjustable thermal expansion properties, in order to seal a large variety of materials, such as metals with comparatively high coefficients of thermal expansion used in high-temperature reactors or materials with lower coefficients of thermal expansion such as glasses or ceramics.

## Methods

Ternary silicates from the solid solution series Ba_1-x_Sr_x_ZnSi_2_O_7_ (0 ≤ × < 1) were synthesized via conventional ceramic mixed oxide route so that 25 g of ceramic powder were obtained. Mixtures of SiO_2_ (Carl Roth GmbH + Co. KG, >99%), BaCO_3_ (VK Labor- und Feinchemikalien, pure), SrCO_3_ (Ferak Berlin, >99%) and ZnO (Carl Roth, >99%) were used as raw materials. The mixtures were heated up to temperatures between 1125 to 1250 °C, kept for 30 to 50 h with several intermediate regrinding steps.

The phase purity was verified by X-ray powder diffraction (XRD), using a SIEMENS D5000 Bragg-Brentano diffractometer and CuK_α_ radiation. The XRD-patterns of the powdered samples were recorded between 10 ≤ 2θ ≤ 60° with a step size of Δθ = 0.02° and a counting time of 1 s per step.

The same device was used for high temperature XRD (HT-XRD). Here, the diffractometer was equipped with an ANTON PAAR HTK 10 heating stage. The powdered samples were heated to the respective temperatures, kept for several minutes until temperature equilibrium is reached and afterwards an XRD scan was performed. The samples were measured in a 2θ range from 10–60°. All the samples, analyzed by HT-XRD contained 33 wt% of α-Al_2_O_3_ as an internal standard. The peak positions and cell parameters of α-Al_2_O_3_ are known from the ICSD database (27 °C: ICSD10425, 300 °C: ICSD160605, 600 °C: ICSD160606, 900 °C: ICSD160607) and agree with other data from the literature^18^,^21^. From these patterns, the cell parameters were calculated with the software TOPAS from Bruker.

Single crystals were prepared using the following procedure: Ba_0.5_Sr_0.5_Zn_2_Si_2_O_7_ ceramic powder was melted in a small platinum crucible (6 mm in diameter and 2 mm in height) at around 1500 °C and slowly cooled down to 1150 °C with a cooling rate of 1 K/min. Afterwards, the sample was cooled fast to room temperature. The so prepared sample was polycrystalline, but contained large single crystalline regions. Single crystals were obtained by mechanically destroying the polycrystals. A polarization microscope was used to identify the single crystals. After single crystal X-ray diffraction, the composition of the crystal was determined using energy dispersive X-ray spectroscopy (EDS). Therefore, a JEOL JSM 7001F electron microscope was used. The composition of the crystal was determined using an acceleration voltage of 30 kV. This high voltage has to be used in order to stimulate the Sr Kα energy at 14.163 keV. Lower values lead to erroneous values because of the strong overlapping of the Kα energy of Si at 1.74 keV and the Lα energy of Sr at 1.806 keV.

Dilatometric measurements were performed between room temperature and 900 °C (heating rate 5 K/min), using a dilatometer NETZSCH Dil 402 PC. The ceramic powders were cold isostatically pressed into cylindrical shape (diameter: 6–8 mm, length: 5–25 mm) and afterwards sintered between 1125 and 1300 °C. The hot pressed sample was prepared by uniaxial pressing with a pressure of 28 MPa, kept for 5 min at 1100 °C.

The single-crystal structure determination was performed as follows. The intensity data for the compounds were collected on a Nonius KappaCCD diffractometer using graphite-monochromated Mo-K_α_ radiation. The data were corrected for Lorentz and polarization effects; absorption was taken into account on a semi-empirical basis using multiple-scans^22^,^23^,^24^.

The structures were solved by direct methods (SHELXS^25^) and refined by full-matrix least squares techniques against F_o_^2^ (SHELXL-97^25^). The Ba^2+^ and Sr^2+^ positions are disordered on the m2 m site symmetry of the space group Cmcm (No. 63). The occupancy factors were refined for the same crystal at different temperatures for Sr^2+^ to 39.8(7) % (220 K), 39.6(7) % (293K), and 40.5(7) % (353 K), respectively. For the final refinement, the site occupancy factors were fixed for all temperatures to 60% for Ba^2+^ and to 40% for Sr^2+^. All atoms were refined anisotropically^25^.

Crystallographic data as well as structure solution and refinement details are summarized in Table 1. DIAMOND 3.2 was used for structure representations.

## Additional Information

**How to cite this article**: Thieme, C. *et al.* Ba_1−x_Sr_x_Zn_2_Si_2_O_7_ - A new family of materials with negative and very high thermal expansion. *Sci. Rep.* **5**, 18040; doi: 10.1038/srep18040 (2015).

## Figures and Tables

**Figure 1:**
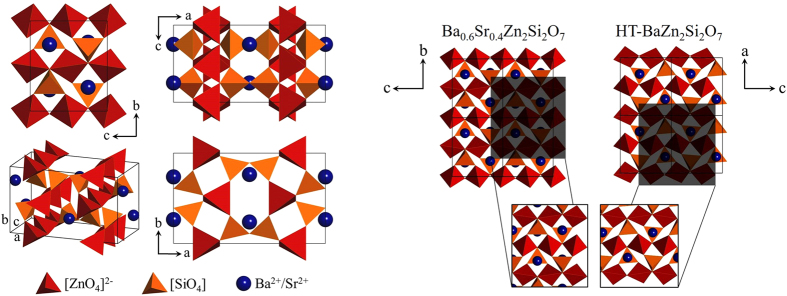
The crystal structure of the Ba_0.6_Sr_0.4_Zn_2_Si_2_O_7_ phase at 20 °C. The left part of the image displays the crystal structure with different views. On the right side, a comparison between the crystal structures of Ba_0.6_Sr_0.4_Zn_2_Si_2_O_7_ and HT-BaZn_2_Si_2_O_7_ is displayed. The blue spheres are the Ba^2+^ and Sr^2+^ ions inside the Ba_0.6_Sr_0.4_Zn_2_Si_2_O_7_ phase. In the case of BaZn_2_Si_2_O_7_, the blue balls only describe the positions of Ba^2+^.

**Figure 2:**
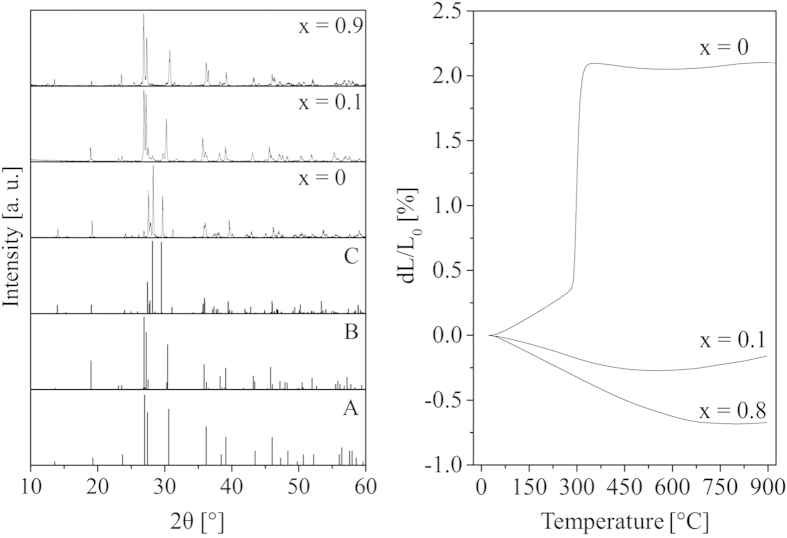
Changes in the crystal structure and the thermal expansion behavior in the solid solution series Ba_1-x_Sr_x_Zn_2_Si_2_O_7_ for different values of x. On the left side, X-ray powder diffraction patterns measured at room temperature are presented. In the lower part of the graph, the peak positions, determined by Klasens *et al.* in 1957^11^ (**A**), those calculated from the crystal structure of a Ba_0.6_Sr_0_._4_Zn_2_Si_2_O_7_ single crystal (**B**) and those of LT-BaZn_2_Si_2_O_7_ taken from ref. 13 (**C**) are given. In the upper part, measured diffractograms with x = 0, 0.1 and 0.9 are shown. On the right side, the thermal expansion behavior, determined via dilatometry, is given for x = 0, 0.1 and 0.8. The curve of the sample with x = 0 was taken from Ref. 10.

**Figure 3:**
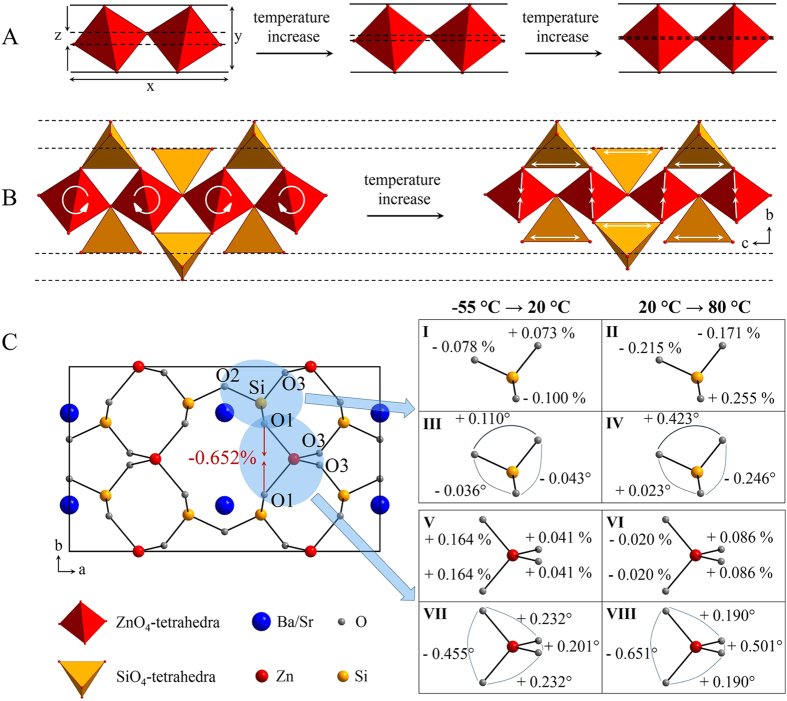
The origin of the negative thermal expansion in crystals with the structure of Ba_0.6_Sr_0.4_Zn_2_Si_2_O_7_. (**A**) Movement of ZnO_4_-tetrahedra in a single chain. (**B**) Rotation and distortion inside the network. (**C**) Distortions of SiO_4_- and ZnO_4_-tetrahedra at different temperatures. The values were taken from single crystal X-ray diffraction measurements. On the left side, the a-b-plane of one unit cell is shown. The red arrows between two O1-atoms show the main direction of shrinkage with a shortening of 0.652% during heating from −55 °C to 80 °C. On the right side, the changes in the O-Si bond length (I, II) and the changes in the O-Si-O bond angles (III, IV) are displayed for two different temperature ranges, given in [%] and [°], respectively. By analogy it was done for the [ZnO_4_]^2^^−^-tetrahedra (V-VIII).

**Figure 4:**
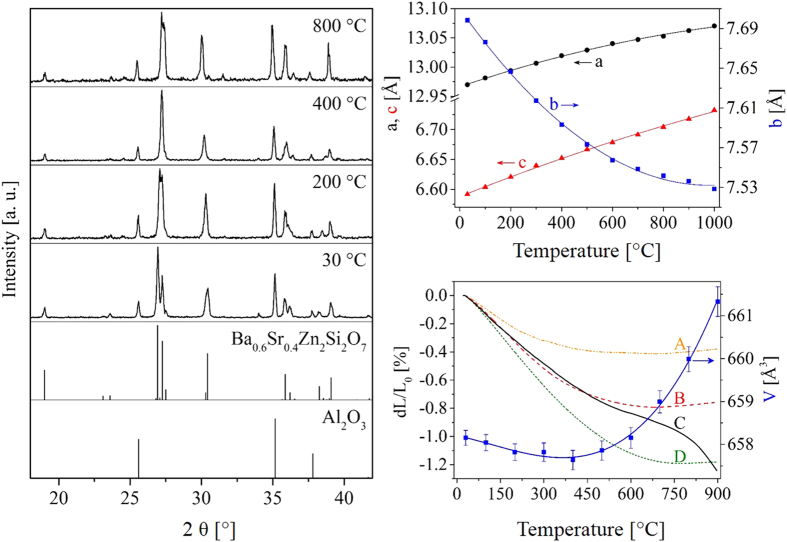
Thermal expansion of Ba_0.6_Sr_0.4_Zn_2_Si_2_O_7_. On the left side, XRD-patterns, recorded at various temperatures are presented in a 2θ range between 18° and 42°. In the lower two diffractograms, the peak positions of Al_2_O_3_ (internal standard) and Ba_0.6_Sr_0.4_Zn_2_Si_2_O_7_ are shown. The peaks of Al_2_O_3_ were taken from ref. 14. In the upper right corner, the cell parameters, determined by X-ray powder diffraction are given as a function of the temperature. In the lower right corner, the dilatometric thermal expansion behavior of bulk ceramics, prepared with different sintering conditions is illustrated: (**A**) sintered at 1300 °C without compression (**B**) isostatically pressed with 100 bar and afterwards sintered at 1175 °C; (**C**) hot pressed at 1100 °C with an uniaxial pressure of 28 MPa; (**D**) sintered at 1270 °C without compression. Furthermore, the volume of the unit cell is shown (blue symbols).

**Table 1:** Crystal data and refinement details for the X-ray structure determinations at different temperatures.

temperature	220 K	293 K	353 K
formula	Ba_0.6_Sr_0.4_Zn_2_Si_2_O_7_	Ba_0.6_Sr_0.4_Zn_2_Si_2_O_7_	Ba_0.6_Sr_0.4_Zn_2_Si_2_O_7_
fw (g∙mol^−1^)	416.41	416.41	416.41
T*/°C*	−55(2)	20(2)	80(2)
crystal system	orthorhombic	orthorhombic	orthorhombic
space group	C m c m	C m c m	C m c m
*a*/ Å	12.9689(6)	12.9792(7)	12.9907(8)
*b*/ Å	7.7251(4)	7.7081(4)	7.6710(5)
*c*/ Å	6.5767(3)	6.5887(4)	6.6148(4)
*V*/Å^3^	658.89(5)	659.17(6)	659.18(7)
*Z*	4	4	4
*ρ* (g∙cm^−3^)	4.193	4.193	4.193
*μ* (cm^−1^)	143.54	143.54	143.54
measured data	2771	2701	2397
data with I > 2σ(I)	497	531	527
unique data (R_int_)	500/0.0356	538/0.0605	539/0.0403
w*R*_2_ (all data, on F^2^)a)	0.0485	0.0537	0.0574
*R*_1_ (*I* > 2σ(*I*))a)	0.0191	0.0227	0.0225
*S*^b)^	1.251	1.217	1.169
Res. dens./e∙Å^−3^	0.711/−1.064	0.733/−1.050	1.214/−1.313
absorpt method	multi-scan	multi-scan	multi-scan
absorpt corr T_min_/_max_	0.5552/0.7458	0.5893/0.7460	0.5631/0.7460
CSD- No.	429937	429938	429939

^a^Definition of the *R* indices: R_1_ = (Σ||*F*_o_| – |*F*_c_||)/Σ |*F*_o_|; wR_2_ = {Σ[*w*(*F*_o_^2^ – *F*_c_^2^)^2^]/Σ[*w*(*F*_o_^2^)^2^]}^1/2^ with *w*^−1^ = σ^2^(*F*_o_^2^) + (*aP*)^2^ + bP; P = [2F_c_^2^ + Max(F_O_^2^]/3;

^b^*s* = {Σ[*w*(*F*_o_^2^ – *F*_c_^2^)^2^]/(*N*_o_ – *N*_p_)}^1/2^.
